# First case report of malignant peritoneal mesothelioma and oral verrucous carcinoma in a patient with a germline *PTEN* mutation: a combination of extremely rare diseases with probable further implications

**DOI:** 10.1186/s12881-018-0651-4

**Published:** 2018-08-15

**Authors:** Markus W. Löffler, Julia Steinhilber, Franz J. Hilke, Sebastian P. Haen, Hans Bösmüller, Ivonne-Aidee Montes-Mojarro, Irina Bonzheim, Antje Stäbler, Ulrike Faust, Ute Grasshoff, Ingmar Königsrainer, Hans-Georg Rammensee, Lothar Kanz, Alfred Königsrainer, Stefan Beckert, Olaf Riess, Christopher Schroeder

**Affiliations:** 1 Department of General, Visceral and Transplant Surgery, University Hospital Tübingen, Hoppe-Seyler-Str. 3, 72076 Tübingen, Germany; 2 Interfaculty Institute for Cell Biology, Department of Immunology, University of Tübingen, Auf der Morgenstelle 15, 72076 Tübingen, Germany; 3 German Cancer Consortium (DKTK) and German Cancer Research Center (DKFZ) partner site Tübingen, Tübingen, Germany; 4 Institute of Pathology and Neuropathology, University Hospital Tübingen, Liebermeisterstr. 8, 72076 Tübingen, Germany; 5 Institute of Medical Genetics and Applied Genomics, University of Tübingen, Calwerstr. 7, 72076 Tübingen, Germany; 6 Internal Medicine, Department for Oncology, Hematology, Immunology, Rheumatology and Pulmonology, University of Tübingen, Otfried-Müller-Str. 10, 72076 Tübingen, Germany

**Keywords:** Malignant Peritoneal Mesothelioma, PTEN-Hamartoma-Tumor-Syndrome, Hereditary Tumor Syndrome, Verrucous Carcinoma, Case Report

## Abstract

**Background:**

The *PTEN*-hamartoma-tumor-syndrome (PHTS) is caused by germline mutations in *Phosphatase and Tensin homolog (PTEN)* and predisposes to the development of several typical malignancies. Whereas *PTEN* mutations have been implicated in the occurrence of malignant mesotheliomas, the genetic landscape of verrucous carcinomas (VC) is largely uncharted. Both VC and malignant peritoneal mesotheliomas (MPM) are exceedingly rare and a potential link between these malignancies and PHTS has never been reported.

**Case presentation:**

We here describe the clinical course of a PHTS patient who, in addition to a typical thyroid carcinoma at the age of 36 years, developed a highly-differentiated oral VC and an epithelioid MPM six years later. The patient with a history of occupational asbestos exposure underwent cytoreductive surgery and hyperthermic intraperitoneal chemotherapy for MPM. The clinical diagnosis of PHTS was consequently corroborated by a germline *PTEN* deletion. Sequencing of tumor tissue revealed a second hit in *PTEN* in the thyroid carcinoma and VC, confirmed by a *PTEN* loss and activation of the PI3K/AKT pathway in immunohistochemistry. Furthermore, additional somatic mutations in the thyroid carcinoma as well as in the VC were detected, whereas the genetics of MPM remained unrevealing.

**Discussion and conclusions:**

We here report the very unusual clinical course of a patient with rare tumors that have a germline mutation first hit in *PTEN* in common. Since this patient was exposed to asbestos and current evidence suggests molecular mechanisms that might render PHTS patients particularly susceptible to mesothelioma, we strongly recommend PHTS patients to avoid even minimal exposure.

**Electronic supplementary material:**

The online version of this article (10.1186/s12881-018-0651-4) contains supplementary material, which is available to authorized users.

## Background

The *PTEN*-hamartoma-tumor-syndrome (PHTS) is a cancer predisposition syndrome with autosomal dominant inheritance, caused by mutations in the *PTEN* (phosphatase and tensin homolog) tumor suppressor gene. PHTS can be sub-classified into four clinical phenotypes, the Cowden-, Bannayan-Riley-Ruvalcaba-, *PTEN*-related Proteus- and Proteus-like syndrome [1]. The clinical picture of Cowden Syndrome (CS) includes common features like multiple hamartomas, distinct mucocutaneous lesions, macrocephaly, cerebellar dysplastic gangliocytoma (Lhermitte-Duclos disease) and typical malignancies, showing nearly complete penetrance by the age of thirty [1]. Mutations in *PTEN* impair its function and result in the stimulation of PI3K-AKT signaling, whereas functional *PTEN* dephosphorylates phosphatidyl-inositolphosphates that inhibit MAP kinase signaling [2] and promote Ca^2+^ mediated apoptosis [3]. This decreases cellular proliferation, transformation and survival of cells with DNA damage. Accordingly, *PTEN* germline mutations predispose to the development of different cancers and coincide with a highly increased life-time risk for specific malignancies. In females, the corresponding risk for breast and endometrium cancer has been estimated at 85 % and 28%, respectively, and a correlation with the incidence of follicular thyroid cancer (35%) is well established [4]. Further, an elevated risk for colorectal (9%) and kidney cancer (34%) as well malignant melanomas (6%) was reported [1, 4]. For mutation carriers, clinical diagnostic criteria catalogues such as screening programs are available, the latter including thyroid ultrasound, dermatological evaluations, breast cancer screening and colonoscopies [1]. The prevalence of CS has been estimated at one per 200,000, but is likely underdiagnosed [1], therefore disease awareness should be raised.

We here report the case of a CS patient with a history of papillary thyroid carcinoma, developing the very unusual combination of malignant peritoneal mesothelioma (MPM) and well-differentiated verrucous (squamous cell) carcinomas (VC) involving the oropharynx and larynx, a co-occurrence of rare malignancies hitherto without precedent. The latter tumors are verrucous affections that constitute a squamous cell neoplasia of uncertain dignity and possibly only a facultative pre-cancerosis also termed Ackerman’s tumor [5]. Etiologically it has been hypothesized that human papilloma viridae (HPV) may be causative for this disease [6] as well as chronic irritants, including tobacco chewing. Such tumors are very rare and represent only a small fraction of oral tumors that have a yearly incidence of only about 1–3 per million persons [7], resulting in barely any information available in the international medical literature.

Additionally, malignant peritoneal mesotheliomas (MPM) are exceedingly rare, with an incidence of only 0.2–3 and 0.5–2 cases per million for women and men, respectively [8]. Histologically MPM can be classified into three subgroups encompassing epithelioid, sarcomatoid and mixed (biphasic) subtypes [9], all usually diagnosed in late stage, due to unspecific symptoms and therefore associated with an unfavorable prognosis [10]. Somatic mutations in *PTEN* have been implicated in the emergence of many different tumor types including pleural mesothelioma, where loss of *PTEN* was described as a frequent event and associated with a worse prognosis [11].

## Case presentation

**Fig. 1:**
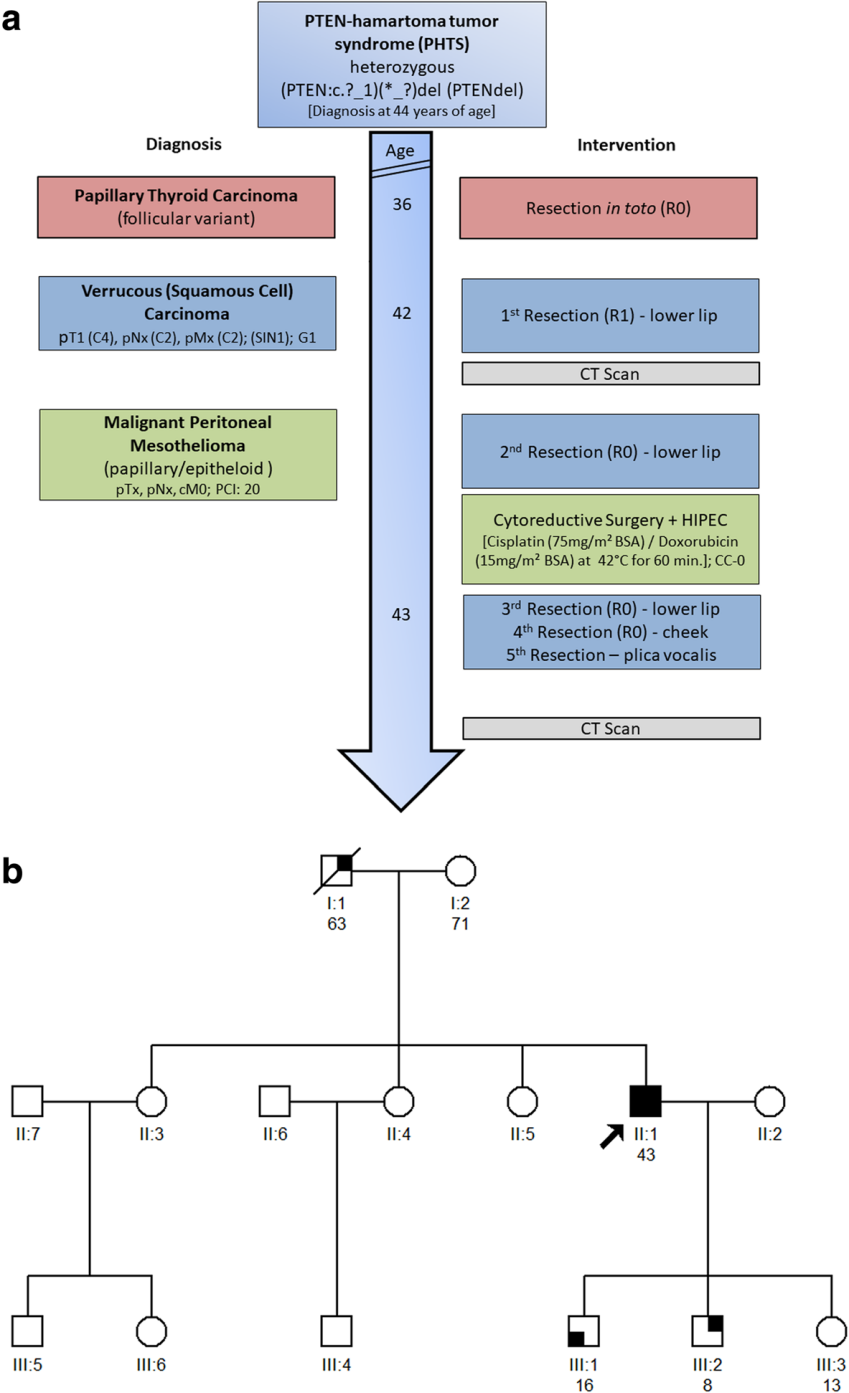
(**a**) Timeline. Relevant events, depicted in the clinical course of the patient (respective age is given in years) with focus on diagnosis and interventions, are represented in a chronological order (time line is not true to scale, colors on both sides correspond to each other). (**b**) Pedigree chart. The index patient presented with macrocephaly, typical mucocutaneous lesions and a history of different tumor diseases. He was diagnosed with thyroid cancer at the age of 36 years and with a verrucous carcinoma (VC) and malignant peritoneal mesothelioma (MPM) at the age of 42 years. The combination of the clinical symptoms subsequently led to the clinical diagnosis of PTEN-hamartoma tumor syndrome (PHTS). The patient stated that one of his sons and his father were also macrocephalic (III:2, I:1). Another son was reported to have cutaneous papillomatosis (III:1). The arrow marks the index patient, squares males and circles females. Filled symbols indicate clinical symptoms that support the diagnosis of PHTS

A Caucasian male patient with a history of a (follicular type) papillary thyroid carcinoma resected in toto at the age of 36 years (Fig. 1a), was scheduled for a staging CT scan after incomplete resection (R1) of a highly-differentiated verrucous (squamous cell) carcinoma (VC) of the lower lip (staged as pT1 (C4), pNx (C2), pMx (C2), G1 (squamous intraepithelial neoplasia SIN1)) at the age of 42 years. In imaging findings, suspect peritoneal lesions consistent with peritoneal metastases from colorectal carcinoma were observed and confirmed as metabolically active by (^18^F)-FDG PET/CT, without any positive mediastinal lymph nodes. However, a colonoscopy and gastroscopy remained without pathological findings. The ensuing diagnostic laparoscopy confirmed disseminated nodular lesions spread over the small bowel, transversal colon and the liver, which were diagnosed as a differentiated epithelioid mesothelioma with papillary growth type (pan-Cytokeratin^(+++)^; Vimentin^(+)^; CK5/6^(+)^; BerEp4^neg.^; GLUT1^neg^; MIB-1^+^ ~ 5%) [12, 13]. Subsequently, the patient was scheduled for cytoreductive surgery (CRS) and hyperthermic intraperitoneal chemotherapy (HIPEC; with Cisplatin (75 mg/m^2^ body surface area)/ Doxorubicin (15 mg/m^2^ body surface area) for 60 min at 42 °C target temperature). During CRS, peritonectomy, omentectomy and ileum-segment resection was performed and the disseminated tumor (PCI score: 20) [14] was macroscopically completely resected (CC-0) [15]. The resected MPM (staged pTx, pNx, cM0) was additionally found to be positive for nuclear WT1^(+)^, membrane-bound podoplanin (D2–40)^(+)^ and calretinin^(+++)^ in immuno-histochemistry [12]. Since an established staging system for peritoneal mesothelioma is lacking, the findings may be classified as a T2 tumor according to a clinicopathological staging system proposed in 2011 [16]. Repeated abdominal and thoracic CT scans remained without signs of recurrence during a two-year follow-up period after CRS and HIPEC (Fig. 1a). The patient’s medical history includes a history of smoking (until the age of 42 years), such as occupational exposure to asbestos and an axial hiatal hernia with gastroesophageal reflux disease (GERD). The patient, a carpenter by profession reported frequent occupational exposure to fibre cement roofing, containing mainly chrysotile asbestos for a period of about seven years, starting 25 years previous to being diagnosed with MPM. He was additionally exposed to asbestos containing industrial flooring in the course of building renovations, during a subsequent 5 years period. A thoracic CT scan confirmed diffuse bipulmonal thickening, appearing as milky spots and nodules remaining of constant size over time, as well as emphysematous alterations of parenchymatous lung tissue, without any lesions suspicious of malignancy. Of note, no gastrointestinal or B symptoms were apparent before diagnosis of MPM. Further, no known history of familial cancer in the parents and the father’s seven siblings was reported, but there was a history of colon cancer and bladder cancer in two of the three brothers of the mother (Fig. 1b). On examination, the patient presented with macrocephaly (head circumference 64 cm) as well as oro-laryngeal and cutaneous papillomatosis. Macrocephaly was also reported in the patient’s father. The patient fathered three children, one of whom was reported to be macrocephalic and a second was diagnosed with papilloma of the skin. Further segregation analysis was recommended. The oro-laryngeal papillomas of the aerodigestive tract showed degeneration into a highly-differentiated verrucous squamous neoplasia and had to be resected on multiple occasions (Fig. 1a), but apart from an extensive acanthosis and papillomatosis of the lesions, these were characterized by missing HPV and fungal infection, such as a low proliferation rate (MIB-1; p16^neg.^) and showed only a discrete lymphocytic infiltrate.

The patient’s history and clinical examination prompted to the possibility of a PHTS being causally involved in the course of the disease and sufficient positive criteria for an operational diagnosis of CS according to the International Cowden Consortium Diagnostic Criteria were present [17]. Respective sequencing and gene dosage analysis of *PTEN* (ENSG171862) confirmed a heterozygous germline deletion of the complete *PTEN* gene (c.(?_1)_(*_?)del), which has already been syndrome associated [18].

To pinpoint additional somatic mutations and to genetically characterize the different tumorous lesions (Fig. 1a), we used a previously described custom cancer panel [19].

**Fig. 2:**
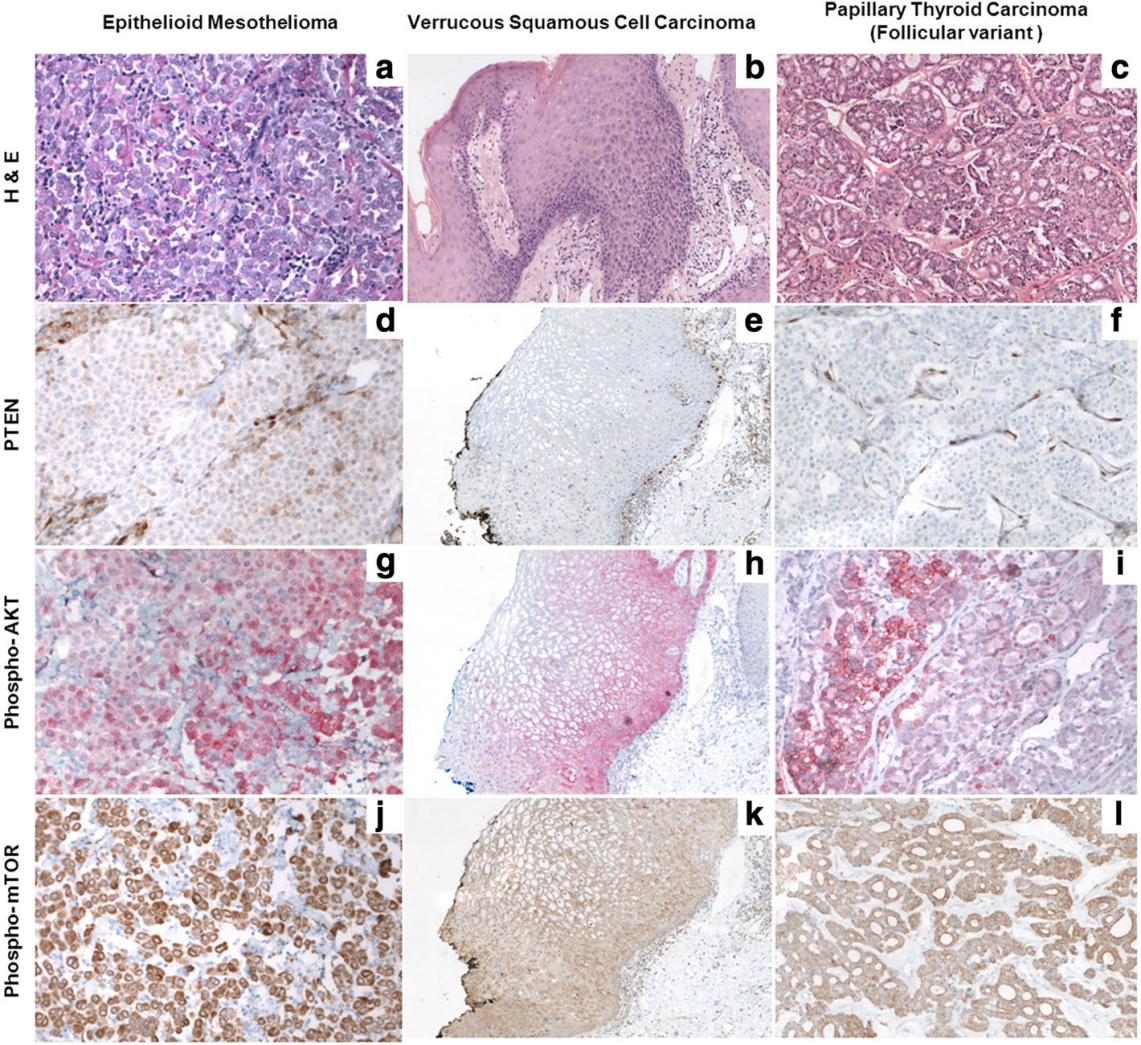
PTEN, Phospho-AKT and Phospho-mTOR staining. H&E staining: epithelioid peritoneal mesothelioma (MPM) (**a**), verrucous (squamous cell) carcinoma (VC) (**b**), and papillary thyroid carcinoma (**c**). PTEN: Staining of respective tumors (stromal cells and vascular structures may serve as positive internal control) (**d**, **e**, and **f**). Phospho-AKT: immunostaining of respective tumors (**g**, **h**, and **i**). Phospho-mTOR: strong cytoplasmic staining in the MPM (**j**), weak cytoplasmic staining in the VC (**k**), and moderate cytoplasmic staining in the thyroid carcinoma **(l)**; (all magnifications: 200×)

In this way, we detected various mutations including *LRP1B* (NM_018557.2:c.2623G > A), *EPHA7* (NM_004440.3:c.2812G > A), *JAK2* (NM_001322194.1:c.614 + 20C > T), *NF1* (NM_001042492.2:c.5764C > T) and one truncating variant in *PTEN* (NM_001304717.2:c.907C > T) in the VC. The genetic profile of the thyroid carcinoma revealed two further classical driver mutations, namely *KMT2D* (NM_003482.3:c.6235-6dupC) and *TP53* (NM_000546.5:c.626_627delGA), confirming the diagnosis on a molecular level. A larger region of loss of heterozygosity indicated a loss of the second *PTEN*-allele also in this tumor. Congruent with previous findings in pleural mesothelioma suggesting their scarcity, we did not identify any somatic oncogene mutations in the MPM [20, 21], although most of the genes previously described as frequently mutated in this malignancy [22] were covered by our panel. Off note, no loss of the second *PTEN-*allele was detected in this sample. With regard to the *PTEN* deletion and its downstream effects, we have analyzed all three tumors by immunohistochemistry (Fig. 2). PTEN staining appears weak but focal positive in MPM and negative in in all other tumors (Fig. 2d-f), whereas skin (Fig. 2e) and vascular structures (Fig. 2f) stained strongly positive, which may be regarded as an internal control. These findings are in line with the sequencing results and confirm the loss of functional PTEN in two out of three tumor specimens. The downstream phospho-AKT staining was found positive in > 90% of malignant cells, at least at basal levels, and resulted strongly positive in up to 40% of neoplastic cells observed in the thyroid carcinoma and MPM. The phospho-mTOR staining corroborated an increased activity of this signaling pathway, showing a strong staining pattern in MPM and a moderate to weak staining pattern in the papillary thyroid carcinoma as well as the VC (Fig. 2j-l). Details on the respective materials and methods used are provided in Additional file 1.

## Discussion and conclusions

The presented case is remarkable regarding several different aspects. Not only is PHTS a very rare condition [23], but so are MPM [8] and highly differentiated VC [7]. It is therefore conceivable that in connection with an established hereditary tumor syndrome such as PHTS, a preexisting papillomatosis of the aerodigestive tract might evolve into a highly differentiated VC, but this has never been reported previously and is purely speculative. The same is generally true for an interconnection between PHTS and MPM, whereas the incidence of thyroid carcinomas is known to be strongly increased in this condition [4], rendering this presented case already special and unique. However, intriguingly another hereditary tumor syndrome affecting the tumor suppressor gene BRCA1-associated protein 1 (*BAP1*) -likewise a potent tumor-suppressor gene- was discovered due to an exceptionally high incidence of mesotheliomas (and uveal melanomas) in two families and linked to asbestos exposure [24]. Further it has been established, that both BAP1 and PTEN have effects on controlling inositol 1,4,5-trisphosphate (IP3) generation and thereby IP3 receptor-mediated mitochondrial Ca^2+^ flux [3, 25]. PTEN on the other hand competes with FBXL2 for IP3 receptor type 3 (IP3R3) binding, preventing the degradation of IP3R3, thereby inhibiting apoptosis [3]. For BAP1^+/−^ carriers it has been shown that decreased deubiquitination and thus IP3R3 stabilization at the endoplasmic reticulum reduces mitochondrial Ca^2+^ flux, preventing apoptosis in cells with DNA damage and therefore resulting in cell survival and increased cellular transformation rates [25]. Interestingly, these findings link environmental stressors to malignancy [26]. Furthermore, in heterozygous BAP1^+/−^ individuals, metabolic alterations have been evidenced, which have been implicated in a large array of cancers and suggested as a hallmark of rapidly proliferating cells [27, 28]. These involve increased aerobic glycolysis and lactate production as well as reduced mitochondrial respiration [28], also called *Warburg effect* [29], which predates malignant development in this case, suggesting that respective metabolic alterations may favor oncogenesis. Since both BAP1 and PTEN inhibit apoptosis in a similar way, increasing the likelihood for pre-malignant cells to survive, it is tempting to speculate that also *PTEN* mutations may contribute to mesothelioma development in carriers of *PTEN* mutations, as demonstrated in animal models carrying germline *BAP1* mutations with even minimal exposure to asbestos [30].

It is noteworthy that in this particular instance we have the rare opportunity to analyze several very uncommon neoplasms from the same patient synoptically and we may speculate that these could be facilitated by the heterozygous *PTEN*^+/−^ deletion observed in the germline, possibly in connection with established noxae. In this context and with the comprehensive knowledge already available for BAP1, this present case raises questions that may be relevant for future research and even lend itself to a patient-centered reverse translation approach [31]. However, it should be duly noted that a case report is not suitable to establish any conclusive evidence or even causality.

At the genomic level, in two of the tumors we confirmed a biallelic affection of *PTEN,* namely a truncating variant on the remaining allele in in the VC and a loss of the second *PTEN*-allele in the thyroid carcinoma. These findings are congruent with immunohistochmistry findings, where weak focal PTEN staining is observed in MPM cells but remains negative for both other tumors. The downstream phospho-AKT staining expectable in return showed positive staining at basal levels in almost all malignant cells from all three tumors assessed, whereas strong staining was limited to a substantial part of neoplastic cells in the thyroid carcinoma and MPM, established as full-fledged malignancies. Furthermore, phospho-mTOR staining was particularly strong in the MPM, establishing an increased signaling in these cells through this pathway. Assessing oncogenic somatic mutations in the different tumors, we detected several driver mutations in the VC including *JAK2*, *NF1* and *PTEN*. Respective findings support the notion that although this tumor is usually considered a borderline tumor [5] and appears as well differentiated histomorphologically with minimal metastatic potential, genetically the tumor qualifies as malignant. We identified also other established driver mutations in the thyroid carcinoma, whereas the analysis of the MPM was unrevealing. In case new treatment considerations would be needed, our findings may support the use of an mTOR inhibitor, which is a clinically well-established class of drugs as a treatment of choice with a broad activity spectrum on the tumors occurring in this patient.

Concluding on this case, we would like to raise awareness for PHTS in general and the clinical appearance of Cowden Syndrome in particular, being a genodermatosis often difficult to recognize and therefore diagnosed (too) late. Although various tumors have been established with high incidences or described in connection with PHTS [4], neither MPM nor VC have been conjointly reported. Although due to the rarity of the malignancies reported there is no conclusive evidence available (yet), a highly increased vulnerability to asbestos as a contributing factor to MPM oncogenesis is plausible and supported by molecular mechanisms [3, 25, 30]. From a preventative point of view, we may conclude that persons with PHTS diagnosis should avoid any, even minimal exposure to asbestos, as it currently cannot be excluded that the risk of developing associated malignancies may be highly increased compared to the general population. Until disproven, it seems reasonable to strongly recommend ensuring a working and domestic living environment free of mineral fibers such as asbestos for PHTS patients.

## Additional file

Additional file 1: Further details on materials and methods used for this case study are provided as a supplement. (PDF 101 kb)

## Abbreviations

(^18^F)-FDG: Fludeoxyglucose
BAP1: BRCA1-associated protein 1
BerEP4: Anti-EpCAM (Epithelial Cell Adhesion Molecule) antibody
BSA: Body Surface Area
C: Certainty Factor
°C: Degree Celsius
c: Clinical (staging according to UICC)
CC: Completeness of Cytoreduction (according to Paul Sugarbaker)
CK: Cytokeratin
CRS: Cytoreductive Surgery
CS: Cowden Syndrome
CT: Computerized Tomography
del: Deletion
FBXL2: F-Box And Leucine Rich Repeat Protein 2
G: Grading (according to UICC)
GERD: Gastroesophageal Reflux Disease
GLUT1: Glucose Transporter 1
HIPEC: Hyperthermic intraperitoneal Chemotherapy
IP3: Inositol 1,4,5-trisphosphate
IP3R3: IP3 receptor type 3
M: Metastasis (stage according to UICC)
MAP: Mitogen-activated Protein Kinase
MIB-1: Anti-Ki-67 antibody [MIB: Molecular Immunology Borstel; KI: Kiel]
MPM: Malignant Peritoneal Mesothelioma
mTOR: Mammalian Target of Rapamycin
N: Nodus lymphoideus (lymph node) (stage according to UICC)
p: Histopathologic (staging according to UICC)
PCI: Peritoneal Carcinomatosis Index
PCR: Polymerase Chain Reaction
PET/CT: Positron emission tomography–computed tomography
PHTS: PTEN-hamartoma-tumor-syndrome
PI3K-AKT: Phosphatidylinositol-3-Kinase and Protein Kinase B
*PTEN*: Phosphatase and Tensin Homolog
R: Residual tumor classification
SIN: Squamous Intraepithelial Neoplasia (according to WHO)
T: Tumor (stage according to UICC)
UICC: Union international contre le cancer
VC: Verrucous (Squamous Cell) Carcinoma
WHO: World Health Organization
WT1: Wilms Tumor Protein
