# Supplementary material for: First case report of malignant peritoneal mesothelioma and oral verrucous carcinoma in a patient with a germline PTEN mutation: a combination of extremely rare diseases with probable further implications
Source: BMC Med Genet. 2018 Aug 15;19:144. doi: 10.1186/s12881-018-0651-4 (PMC6094911; doi:10.1186/s12881-018-0651-4)
Supplement: Supplementary file 1 — Further details on materials and methods used for this case study are provided as a supplement. (PDF 101 kb) [file 12881_2018_651_MOESM1_ESM.pdf]

SUPPLEMENTARY MATERIAL

Additional file 1 to:

First case report of malignant peritoneal mesothelioma and oral verrucous carcinoma in a patient with a germline PTEN mutation: a combination of extremely rare diseases with probable further implications

Markus W. Löffler, Julia Steinhilber, Franz J. Hilke, Sebastian P. Haen, Hans Bösmüller, Ivonne-Aidee Montes-Mojarro, Irina Bonzheim, Antje Stäbler, Ulrike Faust, Ute Grasshoff, Ingmar Königsrainer, Hans-Georg Rammensee, Lothar Kanz, Alfred Königsrainer, Stefan Beckert, Olaf Riess, Christopher Schroeder

BMC Medical Genetics, 2018
https://doi.org/10.1186/s12881-018-0651-4

Extended Materials and Methods

Immunohistochemistry

Formalin-Fixed, Paraffin Embedded (FFPE) tissue was cut in 3-5 µm-thick sections and stained with haematoxylin and eosin (H&E). Immunohistochemistry was performed by automated immunostainer (Roche Ventana Medical Systems, Tucson, AZ) according to manufacturer´s instructions for open procedures with slight modifications. Samples were stained with PTEN (138G6), Phospho-AKT (S473) (D9E) and Phospho-mTOR (4EBP1) antibodies (all from Cell Signaling Technology, Frankfurt a. M., Germany). Appropriate positive and negative controls were employed to confirm the adequacy of the staining. The enhancement, extent and pattern of specific antibody immunostaining within a tissue section were determined by the percentage of cells with positive staining of nucleus, cytoplasm or cell membrane. Sections were inspected at 100x and 400x magnification by an expert pathologist.

Molecular Genetics

According to applicable clinical routines, all coding exons of PTEN (ENSG171862) were amplified by PCR in DNA from EDTA blood and sequenced (ABI 3730xl; Applied Biosystems, Darmstadt, Germany). Gene dosage analysis was performed using MLPA-analysis (Kit P225C1, MRC-Holland, Amsterdam, Netherlands).

Somatic variants in tumors were analyzed as previously described [1]. In brief, DNA was isolated from FFPE shavings and blood. Sample DNA was sequenced with a custom cancer panel (SureSelect XT; Agilent, Waldbronn, Germany) covering 1.566 Mbp of coding sequence of 336 different genes using a HiSeq2500 device (illumina, Eindhoven, Netherlands). Bioinformatic data analysis was performed using the somatic analysis pipeline of the megSAP package (https://github.com/imgag/megSAP, version 0.1-677-g875a6f6) and the ngs-bits package (https://github.com/imgag/ngs-bits, version 2018_04-2-g19faf87). Data analysis included adapter trimming with SeqPurge [2], mapping against the Genome Reference Consortium Human Build 37 with BWA-MEM [3], duplicate removal with samblaster [4], variant calling with stelka [5] and annotation with SnpEff /SnpSift [6, 7] plus vcflib (https://github.com/ekg/vcflib) and dbNFSP [8]. The pipeline was run with modified filter settings (splicing variants +/-20 bp, required min. depth tumor and normal 100x).

References

1. Löffler MW, Chandran PA, Laske K, Schroeder C, Bonzheim I, Walzer M, Hilke FJ, Trautwein N, Kowalewski DJ, Schuster H *et al*: Personalized peptide vaccine-induced immune response associated with long-term survival of a metastatic cholangiocarcinoma patient. *J Hepatol* 2016, 65(4):849-855.
2. Sturm M, Schroeder C, Bauer P: SeqPurge: highly-sensitive adapter trimming for paired-end NGS data. *BMC Bioinformatics* 2016, 17:208.
3. Li H, Durbin R: Fast and accurate short read alignment with Burrows-Wheeler transform. *Bioinformatics* 2009, 25(14):1754-1760.
4. Faust GG, Hall IM: SAMBLASTER: fast duplicate marking and structural variant read extraction. *Bioinformatics* 2014, 30(17):2503-2505.
5. Saunders CT, Wong WS, Swamy S, Becq J, Murray LJ, Cheetham RK: Strelka: accurate somatic small-variant calling from sequenced tumor-normal sample pairs. *Bioinformatics* 2012, 28(14):1811-1817.
6. Cingolani P, Patel VM, Coon M, Nguyen T, Land SJ, Ruden DM, Lu X: Using Drosophila melanogaster as a Model for Genotoxic Chemical Mutational Studies with a New Program, SnpSift. *Front Genet* 2012, 3:35.
7. Cingolani P, Platts A, Wang le L, Coon M, Nguyen T, Wang L, Land SJ, Lu X, Ruden DM: A program for annotating and predicting the effects of single nucleotide polymorphisms, SnpEff: SNPs in the genome of Drosophila melanogaster strain w1118; iso-2; iso-3. *Fly (Austin)* 2012, 6(2):80-92.
8. Liu X, Wu C, Li C, Boerwinkle E: dbNSFP v3.0: A One-Stop Database of Functional Predictions and Annotations for Human Nonsynonymous and Splice-Site SNVs. *Hum Mutat* 2016, 37(3):235-241.
